# Noninvasive cardiac output monitoring in a porcine model using the inspired sinewave technique: a proof-of-concept study

**DOI:** 10.1016/j.bja.2019.02.025

**Published:** 2019-04-04

**Authors:** Richard M. Bruce, Douglas C. Crockett, Anna Morgan, Minh Cong Tran, Federico Formenti, Phi Anh Phan, Andrew D. Farmery

**Affiliations:** 1 Centre for Human and Applied Physiological Sciences, School of Basic and Medical Sciences, King's College London, London, UK; 2 Nuffield Department of Clinical Neurosciences, University of Oxford, Oxford, UK; 3 Department of Biomechanics, University of Nebraska, Omaha, NE, USA

**Keywords:** cardiac output, haemodynamic, mechanical ventilation, monitoring, nitrous oxide, noninvasive, respiration, thermodilution

## Abstract

**Background:**

Cardiac output $\left(\dot{Q}\right)$ monitoring can support the management of high-risk surgical patients, but the pulmonary artery catheterisation required by the current ‘gold standard’—bolus thermodilution $\left({\dot{Q}}_{T}\right)$—has the potential to cause life-threatening complications. We present a novel noninvasive and fully automated method that uses the inspired sinewave technique to continuously monitor cardiac output $\left({\dot{Q}}_{\text{IST}}\right)$.

**Methods:**

Over successive breaths the inspired nitrous oxide (N_2_O) concentration was forced to oscillate sinusoidally with a fixed mean (4%), amplitude (3%), and period (60 s). ${\dot{Q}}_{\text{IST}}$ was determined in a single-compartment tidal ventilation lung model that used the resulting amplitude/phase of the expired N_2_O sinewave. The agreement and trending ability of ${\dot{Q}}_{\text{IST}}$ were compared with ${\dot{Q}}_{T}$ during pharmacologically induced haemodynamic changes, before and after repeated lung lavages, in eight anaesthetised pigs.

**Results:**

Before lung lavage, changes in ${\dot{Q}}_{\text{IST}}$ and ${\dot{Q}}_{T}$ from baseline had a mean bias of –0.52 L min^−1^ (95% confidence interval [CI], –0.41 to –0.63). The concordance between ${\dot{Q}}_{\text{IST}}$ and ${\dot{Q}}_{T}$ was 92.5% as assessed by four-quadrant analysis, and polar plot analysis revealed a mean angular bias of 5.98° (95% CI, –24.4°–36.3°). After lung lavage, concordance was slightly reduced (89.4%), and the mean angular bias widened to 21.8° (–4.2°, 47.6°). Impaired trending ability correlated with shunt fraction (*r*=0.79, *P*<0.05).

**Conclusions:**

The inspired sinewave technique provides continuous and noninvasive monitoring of cardiac output, with a ‘marginal–good’ trending ability compared with cardiac output based on thermodilution. However, the trending ability can be reduced with increasing shunt fraction, such as in acute lung injury.

**Editor's key points**

• The inspired sinewave method can be used to determine cardiac output noninvasively in mechanically ventilated subjects.
• This proof-of-concept study aimed to determine the level of agreement and trending ability of this technique with thermodilution-based cardiac output in a pig model.
• Under control conditions, there was a good level of agreement but with wide limits of agreements between both methods, with marginal–good trending ability.
• The trending ability was reduced by lung lavage-induced acute lung injury.
• Further studies should reveal whether inspired sinewave technology could be developed as an accurate cardiac output monitoring technique.

Cardiac output monitoring can be used to manage surgical patients at high risk of haemorrhage or haemodynamic instability, and is an essential part of goal-directed therapy.^1^, ^2^, ^3^ Bolus thermodilution remains the current ‘gold standard’ for measuring cardiac output, but the required pulmonary artery catheterisation is highly invasive and has the potential to lead to life-threatening complications.^4^ Indeed, it is still uncertain whether its application in critically ill patients improves clinical outcomes.^5^, ^6^, ^7^ As such, the use of pulmonary artery catheterisation has declined in favour of techniques that are less invasive, such as transpulmonary thermodilution and those utilising transoesophageal Doppler and pulse contour analysis.^8^

The ideal cardiac output monitor should be noninvasive and automated, and generate continual, accurate, and precise measurements. Measuring cardiac output via a respiratory based method such as the inspired sinewave technique (IST) has the potential to meet these criteria. The theoretical and experimental basis for the IST originates from the work of Zwart and colleagues^9^ and Hahn and colleagues,^10^, ^11^ whereby the concentration of a tracer gas (e.g. nitrous oxide [N_2_O]) is sinusoidally oscillated in inspired air. A mathematical model of the lung processes the resultant amplitude/phase of the expired sinewave signal and recovers values for cardiopulmonary variables such alveolar volume and pulmonary blood flow $\left({\dot{Q}}_{\text{IST}}\right)$.^12^, ^13^, ^14^

This proof-of-concept study aimed at validating the ${\dot{Q}}_{\text{IST}}$ technique in anaesthetised, mechanically ventilated pigs during pharmacologically induced haemodynamic changes and a saline lavage model of acute lung injury. We particularly examined the trending ability and agreement of ${\dot{Q}}_{\text{IST}}$ with invasive cardiac output measurements based on thermodilution.

## Methods

### Inspired sinewave technique

**Fig 1:**
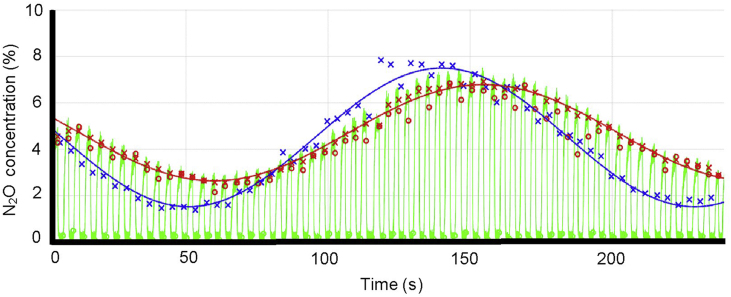
A typical example of recorded data, with inspired N_2_O concentration oscillating sinusoidally around a fixed mean (4%), with a predetermined amplitude (3%) and period (180 s). Green line is the expired N_2_O concentration; the blue and red crosses are the N_2_O concentrations in inspired gas and end-tidal gas, respectively; the blue and red lines are the inspired and expired N_2_O sinewaves, respectively (reproduced from Bruce and colleagues^15^).

Details of the IST and inspired sinewave device (ISD) have been detailed elsewhere,^15^ but in brief: at the start of each inhalation a small volume of N_2_O is injected into inspired gas by a mass flow controller (Alicat Scientific, Inc., Tucson, AZ, USA). Airflow is measured using an ultrasonic flowmeter (VenThor 22/2A; Thor Laboratories, Budapest, Hungary), and N_2_O concentration using a rapid mainstream infrared sensor (Square One Technology, Boulder, CO, USA). The volume injected is proportional to inspiratory flow, and over time (i.e. successive breaths) the inspired N_2_O concentration oscillates sinusoidally around a fixed mean (4%), with a predetermined amplitude (3%) and period (60 s). Without venous recirculation of the N_2_O sinewave, which is assumed negligible at short periods (i.e. <3 min),^16^ the end-tidal N_2_O concentration also oscillate in a sinewave pattern (Fig. 1). A single-compartment tidal ventilation model of the lung uses this resulting amplitude and phase of the expired sinewave to estimate cardiopulmonary variables such as pulmonary blood flow $\left({\dot{Q}}_{\text{IST}}\right)$. The mathematical principles of the IST and the hardware layout of the ISD are provided in Supplementary Fig. S1.

### Animal preparation

Measurements were made in eight female large white pigs with a weight of 37–53 kg (mean=43.6 kg). All procedures in the study were approved by a local ethics committee and the UK Home Office, London, UK (PPL: 30/2960) and performed at the school of Veterinary Sciences, University of Bristol, Bristol, UK. At the end of each study, the animals were killed under anaesthesia with an overdose of pentobarbital (∼100 mg kg^−1^). Relevant sections of the Animal Research: Reporting of *In Vivo* Experiments (ARRIVE) guidelines were followed.

All procedures have been detailed elsewhere,^17^ but in brief: the pigs lay in dorsal recumbency and received i.v. anaesthesia using ketamine, fentanyl, and midazolam. The trachea was intubated, and ventilation was provided in volume-controlled mode using an Engstrӧm Carestation Ventilator (GE Healthcare, Madison, WI, USA), which delivered a tidal volume of 8 ml kg^−1^, with positive end-expiratory pressure set at 5 cm H_2_O. Ventilatory frequency was adjusted to maintain PaCO_2_ between 4.5 and 8 kPa, and it then remained fixed throughout the protocol. Adequacy of general anaesthesia was established with the absence of movement/reflexes the absence of a reaction to painful stimulation between the front hooves, and haemodynamic monitoring. Pancuronium was then administered for muscle relaxation, and Hartmann's solution (10 ml kg^−1^ h^−1^) was used for fluid maintenance. Additional boluses of the muscle relaxant were administered when spontaneous ventilatory efforts were detected from the airway gas and pressure traces. The adequacy of anaesthesia was determined during the periods of muscle relaxation by the absence of cardiovascular signs of sympathetic stimulation (increases in heart rate or arterial BP). Anaesthesia was maintained with continuous i.v. ketamine 32 mg kg^-1^ hr^-1^, fentanyl 4 mg kg^-1^ hr^-1^, and midazolam 0.16 mg kg^-1^ hr^-1^.

A pulmonary artery catheter (PAC; Edward Lifesciences, Irvine, CA, USA) was inserted into the internal jugular vein and advanced into the pulmonary artery. Assessment of pulmonary artery pressure (PAP) and wedge waveforms confirmed the correct position of the PAC. Arterial pressure (via a peripheral artery), PAP, ECG, %SO_2_, and end-tidal CO_2_ were continuously recorded, whereas ${\dot{Q}}_{T}$ was measured intermittently (see below). Variables were monitored with standard patient monitors (Datex Ohmeda Capnomac Ultima; multi-parameter patient monitor, Datex AS3, Madison, WI, USA) and recorded on a computer via PowerLab and LabChart (AD Instruments, Dunedin, New Zealand).

### Experimental protocol

After a stabilisation period of approximately 20 min, baseline measurements were recorded for 10 min, where ${\dot{Q}}_{T}$ measurements did not fluctuate by more than 15%. Cardiac output was then pharmacologically manipulated via infusions of esmolol (300–400 μg kg^−1^ min^−1^), reducing ${\dot{Q}}_{T}$ by at least 1 L min^−1^, followed by infusions of dobutamine (15–20 μg kg^−1^ min^−1^) increasing ${\dot{Q}}_{T}$ by at least 3 L min^−1^ above baseline measurements.

Measurements of ${\dot{Q}}_{\text{IST}}$ are fully automated and continued throughout the entire protocol. ${\dot{Q}}_{\text{IST}}$ is calculated every 30 s as a rolling average of the previous 3 min. ${\dot{Q}}_{T}$ was calculated using the Datex cardiac output module, and was obtained as the average of three 10 ml injections of normal saline through the PAC. ${\dot{Q}}_{T}$ measurements were obtained every 3–5 min, and only three measurements ≤10% apart were used to form the average. As each ${\dot{Q}}_{\text{IST}}$ measurement is a rolling average, linear regression analysis between paired ${\dot{Q}}_{T}$ and ${\dot{Q}}_{\text{IST}}$ measurements was used to determine any time delay, as described elsewhere.^18^ In these experiments, the time delay of ${\dot{Q}}_{\text{IST}}$ measurements was shown to be around 60 s. After correction, paired cardiac output measurements were used for the subsequent analysis.

After these measurements, repeated warm saline lavages were performed (30 ml kg^−1^ 0.9% NaCl at approximately 38°C) until a PaO_2_/F_I_O_2_ ratio of 200–300 mm Hg was achieved, as determined from arterial blood gas analysis. The above experimental protocol was then repeated.

### Statistical analysis

The experiments were designed in accordance with Home Office principles of RRR, essentially minimising animal use whilst preserving data fidelity. Sample sizes were estimated using Mead’s resource equation, as is typical for laboratory animal studies. We chose the sample size to give a value of E (the error degree of freedom) between 10 and 20. Since we used the same animals for measurements before and after lung injury (paired), and a quasi-factorial ‘treatment’ design (i.e. changing cardiac output from resting to higher to lower), a sample size of 8 animals yields an E value of 20. Results are displayed as means 95% confidence interval unless otherwise stated. The agreement of paired absolute ${\dot{Q}}_{T}$ and ${\dot{Q}}_{\text{IST}}$ values were assessed via calculating their interchangeability rate.^19^ Values were considered interchangeable if the difference was less than a maximal acceptable value:

$$\frac{{\dot{Q}}_{\text{T}}+\;{\dot{Q}}_{\text{IST}}}{2}\;\;\text{x}\;\sqrt{R{\dot{Q}}_{T}^{2}+R{\dot{Q}}_{T}^{2}},$$

where RQ_T_ is the repeatability coefficient for ${\dot{Q}}_{T}$ measurements. The agreement between absolute ${\dot{Q}}_{T}$ and ${\dot{Q}}_{\text{IST}}$, and between the changes in ${\dot{Q}}_{T}$ and ${\dot{Q}}_{\text{IST}}$ from baseline ($\Delta {\dot{Q}}_{T}$ and $\Delta {\dot{Q}}_{\text{IST}}$, respectively) were examined via Bland–Altman analysis, and adjusted for multiple measurements within subjects.^20^ The trending ability of ${\dot{Q}}_{\text{IST}}$ *vs* ${\dot{Q}}_{T}$ was examined by calculating the Pearson correlation coefficient (*r*) for paired $\Delta \dot{Q}$ from the previous paired measurements and assessing the concordance (% of paired $\Delta \dot{Q}$ with same directional change) using a four-quadrant plot. A concordance >92% is considered a good trending ability.^21^, ^22^ In addition to assessing the agreement of the direction of $\Delta \dot{Q}$ with a four-quadrant plot, the agreement of the magnitude of $\Delta \dot{Q}$ has been examined with a half-circle polar plot—both calculated with an exclusion zone of 15% mean $\dot{Q}$.^18^, ^21^, ^22^ With polar plots, the radial length of each data point represents the mean of paired $\Delta {\dot{Q}}_{T}$ and $\Delta {\dot{Q}}_{\text{IST}}$, and the polar angle (°) signifies the agreement of the magnitude of $\Delta \dot{Q}$ between the two methods. A mean polar angle (angular bias) of approximately ±5° with a concordance of >95% (% data points within ±30°) (radial limits of agreement) is considered to have a good trending ability, and a concordance of 90–95% is considered marginal.^21^

Trending ability was also assessed via the trend interchangeability method (TIM).^23^ First, changes in ${\dot{Q}}_{T}$ are determined as ‘interpretable’ if no overlap exists between the confidence intervals $\left({\dot{Q}}_{T}\pm {\dot{Q}}_{T}\text{×repeatability}\;\text{coefficient}\right)$ of consecutive measurements. Each interpretable $\Delta {\dot{Q}}_{T}$ is then either determined as interchangeable with $\Delta {\dot{Q}}_{\text{IST}}$, non-interchangeable, or situated within an uncertain interchangeable zone.^23^ Changes are regarded interchangeable if the second pair of measurements (${\dot{Q}}_{T}$ and ${\dot{Q}}_{\text{IST}}$) are found within a predicted precision interval (i.e. within confidence intervals of interchangeable changes), calculated via a predicted line of identity from the first pair of measurements (${\dot{Q}}_{T1}$ and ${\dot{Q}}_{\text{IST}1}$) and the repeatability coefficient of ${\dot{Q}}_{T}$_._ These confidence intervals can be defined as follows: $\text{X}=\text{Y}\left(1+\;\text{RQT}\right)+\left(1+\text{RQT}\right)\left({\dot{Q}}_{T1}-{\dot{Q}}_{\text{IST}1}\right)\;\text{and}\;\text{X}=\text{Y}\left(1-\text{RQT}\right)+\left(1+\text{RQT}\right)\left({\dot{Q}}_{T1}-{\dot{Q}}_{\text{IST}1}\right)$. $\Delta \dot{Q}$ is considered uncertain if its interval of precision intersects one of the confidence intervals, but the value itself does not lie within them.

Differences between baseline variables were assessed via two-tailed unpaired Student's *t*-tests, with statistical significance set at *P*<0.05. All analyses were completed using standard statistical software (Sigma plot version 13; Systat Software, Erkrath, Germany).

## Results

### Uninjured lung

**Fig 2:**
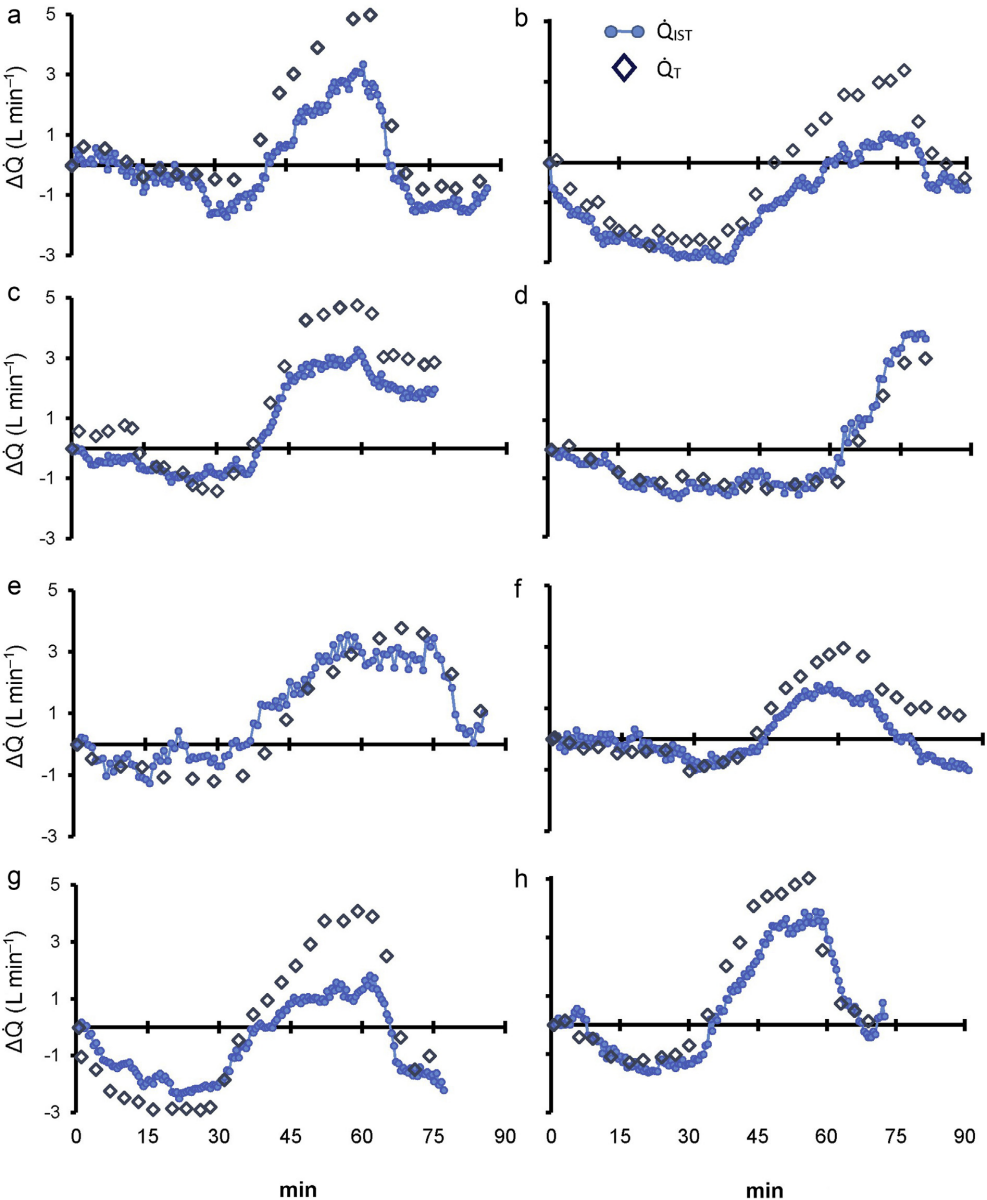
(a–h) Δ$\dot{Q}$ from baseline in all eight animals throughout the protocol. Dark blue diamonds are ${\dot{Q}}_{T}$ measurements, and light blue dots and lines are $\dot{Q}$_IST_ measurements.

**Fig 3:**
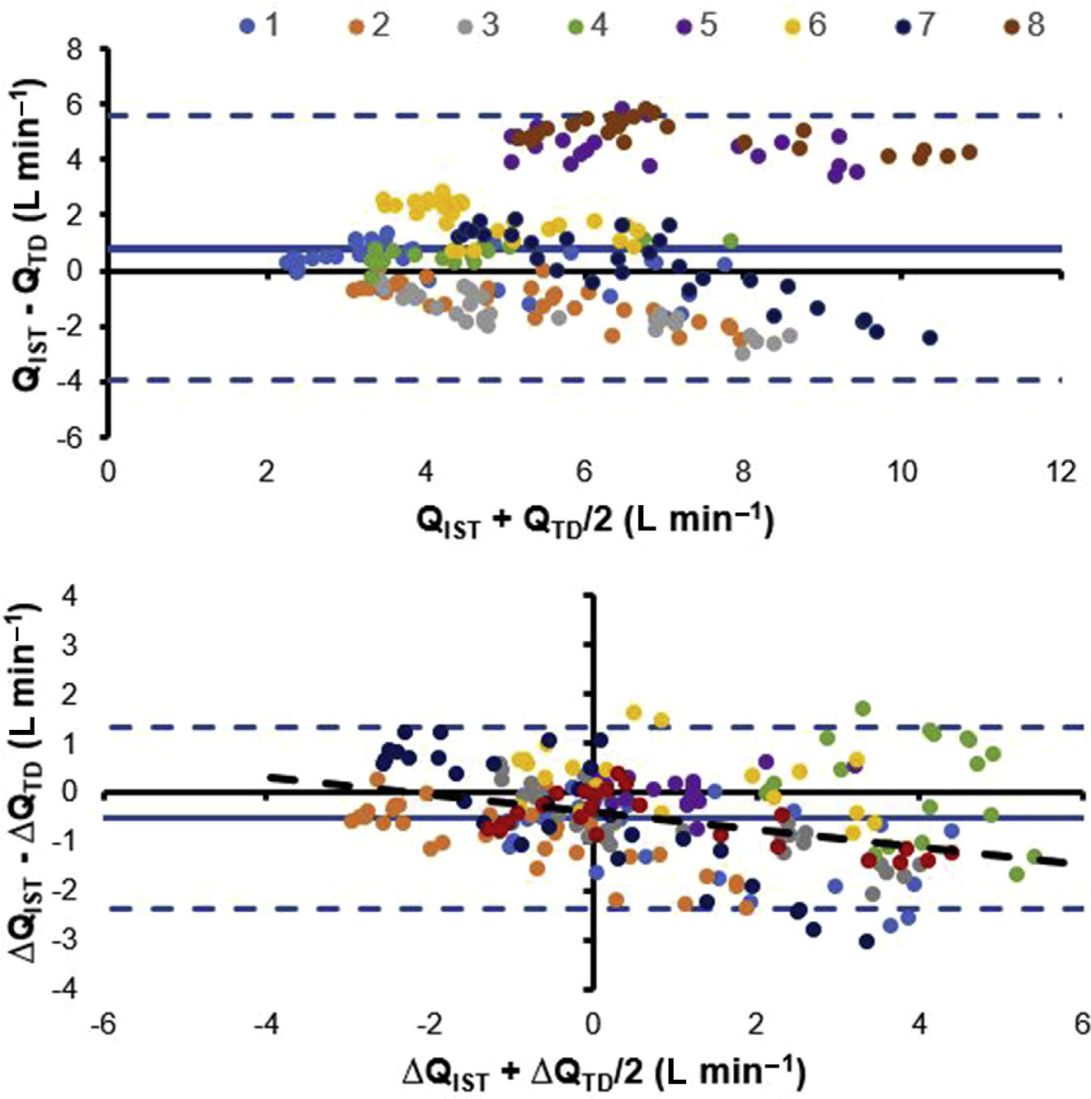
Bland–Altman analysis of the agreement between paired measurements of (a) $\dot{Q}$_IST_*vs*${\dot{Q}}_{T}$ and (b) Δ$\dot{Q}$_IST_*vs* Δ${\dot{Q}}_{T}$ from baseline. Colours represent each animal (1–8). Solid line, mean difference (bias); dotted lines, limits of agreement (LOA).

Baseline haemodynamic and blood gas measurements for each animal are shown in Table 1. All animals survived the protocol, which resulted in marked haemodynamic changes (Fig. 2). A total of 248 paired ${\dot{Q}}_{\text{IST}}$ and ${\dot{Q}}_{T}$ measurements were recorded and the $\Delta \dot{Q}$ values from baseline are presented in Figure 2a–h. Linear regression analysis produced the regression equation $\Delta {\dot{Q}}_{\text{IST}}=0.68\text{×}\Delta {\dot{Q}}_{T}-0.45$, with *r*=0.88. Bland–Altman analysis of absolute ${\dot{Q}}_{T}$ and ${\dot{Q}}_{\text{IST}}$ values (Fig. 3a) shows a mean bias of 0.79 L min^−1^ (0.51, 1.07) with limits of agreement (LOA) ranging from 5.5 (4.6, 6.4) to –3.9 L min^−1^ (–2.8, –4.7). Despite this wide LOA *between* data points from all animals, the LOA *within* each animal is substantially narrower: the LOA for each animal ranged between 0.31 and 0.8 L min^−1^ (mean=0.61 L min^−1^). This is reflected in the Bland–Altman analysis of $\Delta {\dot{Q}}_{\text{IST}}$ *vs* $\Delta {\dot{Q}}_{T}$ (Fig. 3b), where data were normalised to the individual mean baseline value, showing a mean bias of –0.52 (–0.41, –0.63) with LOA ranging from –2.4 (–2, –2.6) to 1.3 (0.9, 2.6), and linear regression analysis reveals an equation of $\Delta {\dot{Q}}_{\text{IST}}-\Delta {\dot{Q}}_{T}=0.27\text{×mean}\;\Delta \dot{Q}-0.56$, *r*=0.48. The interchangeability rate was calculated as 41%. As only poor interchangeability was observed, estimating the range of interchangeable measurements was inappropriate.

**Table 1:** Baseline and respiratory and haemodynamic variables from each animal before repeated saline lavages. DBP, diastolic blood pressure; F_I_O_2_, fraction of inspired O_2_; Hb, haemoglobin; HR, heart rate; IST, inspired sinewave technique; ${\dot{Q}}_{T}$, cardiac output from PAC thermodilution; $\dot{Q}$_IST_, cardiac output from IST; PAC, pulmonary artery catheter; PaCO_2_, arterial CO_2_ partial pressure; PaO_2_, arterial O_2_ partial pressure; PAP, mean pulmonary artery pressure; Paw Peak, peak airway pressure; PFR, PaO_2_/FiO_2_ ratio; SaO_2_, arterial oxygen saturation; SBP, systolic blood pressure

Uninjured Parameter	Animal number								Mean
	1	2	3	4	5	6	7	8	
Weight (kg)	46.5	45	39	53	37	37	47	44.5	43.6
HR (beats min^−1^)	75	70	100	87	60	87	70	102	81
SBP (mm Hg)	113	101	105	80	130	95	105	95	103
DBP (mm Hg)	87	62	71	60	90	85	75	65	74
${\dot{Q}}_{T}$ (L min^−1^)	2.9	6.2	5	4.4	3.9	3.2	6.7	3.7	4.5
$\dot{Q}$_IST_ (L min^−1^)	3.9	6	4.1	4.9	8.1	5.6	7.2	9.2	6.1*
PAP (mm Hg)	20	16	18	15	17	18	16	21	18
Haemoglobin (g dl^−1^)	7.8	8.2	8.9	8.3	10.2	8.3	8.8	9.6	8.8
F_I_O_2_	0.6	0.28	0.4	0.4	0.35	0.4	0.4	0.4	0.4
SaO_2_ (%)	99	99	100	99	95	97	100	97	98
pH	7.37	7.48	7.49	7.38	7.31	7.33	7.33	7.24	7.36
PaO_2_ (mm Hg)	304	115	163	160	186	196	164	175	182
PaCO_2_ (mm Hg)	59	45	44	46	60	56	58	60	53.5
PFR	507	411	408	400	531	490	410	438	449
Paw peak (cm H_2_0)	15	14	16	18	16	16	15	13	15

* significant difference from ${\dot{Q}}_{T}$ (P<0.05)

**Fig 4:**
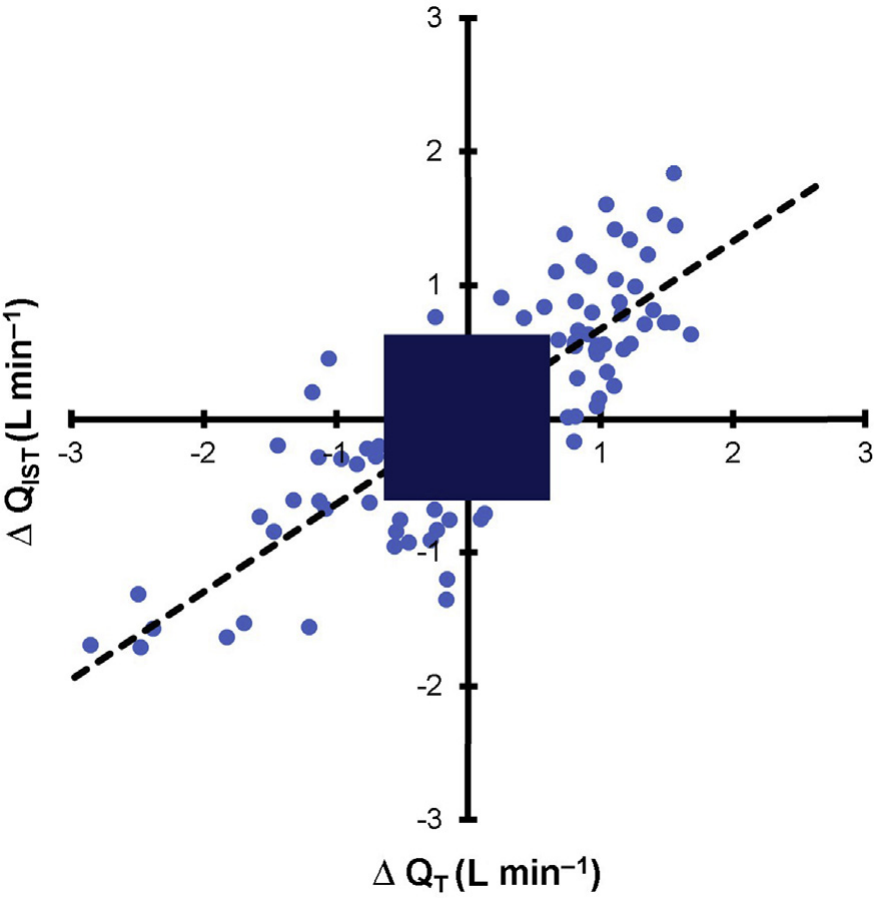
Four-quadrant plot analysis of Δ$\dot{Q}$_IST_*vs* Δ${\dot{Q}}_{T}$ throughout the protocol, with an exclusion zone of 15% mean $\dot{Q}$ (0.67 L min^−1^). Linear regression analysis reveals an equation of Δ$\dot{Q}$_IST_=0.65×Δ${\dot{Q}}_{T}$+0.01, where *r*=0.84. Data points located in either quadrant of agreement were considered concordant (92.5%).

**Fig 5:**
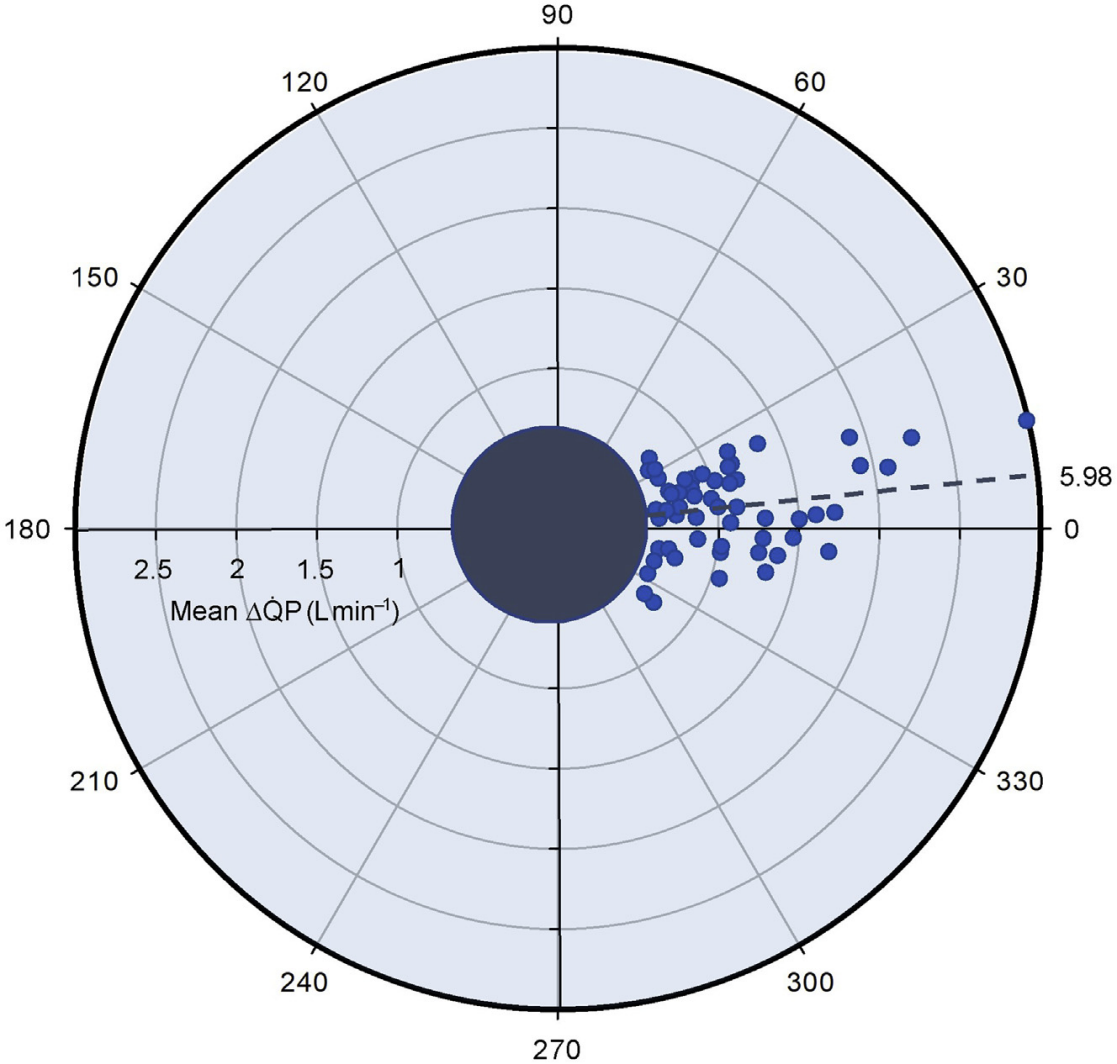
Half-circle polar-plot analysis of $\dot{Q}$_IST_*vs*${\dot{Q}}_{T}$ throughout the protocol, with an exclusion zone of 15% mean cardiac output (0.67 L min^−1^). Mean angular bias=5.98° (radial limits, ±30.2°). Data points located within ±30° limits were considered concordant (92.3%). Data points distributed near the polar axis (0°) indicate good trending.

Trending ability was assessed by calculating the $\Delta \dot{Q}$ from the previous paired measurement values throughout the protocol, with $\Delta \dot{Q}$ <15% mean $\dot{Q}$ excluded. Linear regression shows a high correlation between $\Delta {\dot{Q}}_{\text{IST}}$ and $\Delta {\dot{Q}}_{T}$ of 0.84 (*P*<0.01). The concordance was 92.5% as assessed using four-quadrant analysis (Fig. 4), where data points located in either quadrant of agreement were considered concordant. Linear regression analysis of the four-quadrant plot shows an equation of $\Delta {\dot{Q}}_{\text{IST}}=0.65\text{×}\Delta {\dot{Q}}_{T}+0.01$. Half-circle polar plot analysis (Fig. 5) revealed an angular bias of 5.98° (–24.4°, 36.3°), and a concordance of 92.3% (% of data points within ±30° limits). Half-circle polar plot analysis of the %$\Delta \dot{Q}$ (Supplementary Fig. S3) revealed an angular bias of 4.75° (26.8, 36.2) and a concordance of 90.5%. Trending ability assessed via the TIM demonstrated the following: 65.1% of paired measurements were uninterpretable, 13.2% interchangeable, 9.7% uncertain, and 12% non-interchangeable.

### Injured lung model

Repeated saline lavages resulted in significant reductions in the mean baseline PaO_2_/F_I_O_2_ ratio (250.4 mm Hg [201.4, 299.4] *vs* 449 mm Hg [413, 455]; *P*<0.05). Trending ability was examined using the same approaches discussed above (Supplementary Figs S4 and S5). Four-quadrant analysis revealed a concordance of 89.4%, and polar plot analysis showed a mean angular bias of 21.8° (–4.2°, 47.6°) and a concordance of 69.7%. There was a reduced slope in the linear regression equation of the four-quadrant plot $\left(\Delta {\dot{Q}}_{\text{IST}}=0.3\text{×}\Delta {\dot{Q}}_{T}+0.1\right)$, highlighting that impairments in trending ability after lung injury was the result of underestimations in $\Delta {\dot{Q}}_{\text{IST}}$ relative to $\Delta {\dot{Q}}_{T}$. This impaired trending ability was correlated with shunt fraction (Supplementary Fig. S6; *r*=0.79, *P*<0.05). The $\Delta \dot{Q}$ from baseline in the injured animals are presented in Supplementary Figure S7a–h. Trending ability assessed via TIM demonstrated: 53.7% of paired measurements were uninterpretable, 7.1% interchangeable, 7.6% uncertain, and 31.5% non-interchangeable.

## Discussion

This proof-of-concept study investigated a novel, noninvasive, and fully automated method to continuously monitor changes in cardiac output using the IST. We showed that a prototype device that utilised the IST could be integrated in a breathing circuit of mechanical ventilator, suggesting that it is feasible to use within the perioperative setting. We further demonstrated that a cardiac output based on the IST demonstrated a ‘marginal–good’ trending ability, based upon four-quadrant plot and polar analysis, compared with the pulmonary artery catheter thermodilution reference method.^21^

Thermodilution is widely considered the gold standard technique for cardiac output monitoring. However, because of its highly invasive nature and the associated complications, its clinical use is becoming less frequent.^6^, ^8^ In contrast, the use of less invasive techniques such as those utilising pulse contour analysis or Doppler ultrasound have increased, yet they remain dependent on the skill of the user and their application still requires a somewhat invasive procedure such as an arterial line^8^ (although arterial vascular access is often routinely required in critically ill patients). Respiratory techniques, such as the IST, have the potential to provide an entirely noninvasive method to monitor cardiac output. The IST also has other qualities of an idealised cardiac output monitor,^24^ such as its ability to deliver automated and continual measurements that are independent from the skill of the operator. In addition the IST, like other respiratory-based methods,^25^ can simultaneously estimate other aspects of cardiopulmonary function such as effective lung volume,^15^ a parameter which may provide additional clinically important information for mechanically ventilated patients.

The trending ability of the IST compares well with other noninvasive or minimally invasive methods of monitoring cardiac output. Examining concordance using a four-quadrant plot against thermodilution has been commonly used. The concordance of >92% in the current investigation is commonly regarded as a ‘good’ or clinically acceptable trending ability,^21^, ^22^ and this is similar to that observed in human participants with transoesophageal Doppler^26^, ^27^ and certain pulse contour techniques,^28^, ^29^ and is superior to non-calibrated pulse contour.^30^, ^31^ Furthermore, analysis via TIM revealed a similar percentage of paired interchangeable measurements in comparison with pulse contour techniques such LiDCO and PiCCO.^23^

The findings are also similar to other recently developed respiratory-based approaches utilising the Fick principle,^32^, ^33^ the trending ability of which have also been examined in pigs. In the current investigation polar plot analysis revealed a mean angular bias that was comparable with other respiratory-based methods,^32^, ^33^ and a concordance that was similar to other respiratory-based techniques and considered a ‘marginal’ trending ability.^21^ Altogether, IST seems to have a marginal–good trending ability relative to PAC thermodilution, and is comparable with other respiratory-based or minimally invasive techniques.

Despite the encouraging trending ability, the absolute agreement between ${\dot{Q}}_{\text{IST}}$ and ${\dot{Q}}_{T}$ was weaker. This can be easily observed from the examination of the baseline variables where offset errors have occurred, and from the poor interchangeability rate. Bland–Altman analysis of absolute values revealed an acceptable bias. A wide LOA could make it impossible to achieve good trending ability between $\Delta {\dot{Q}}_{\text{IST}}$ and $\Delta {\dot{Q}}_{T}$, but a closer inspection reveals that the majority of bias variability occurs *between* animals, not *within* each animal. Indeed, the LOA for each individual animal was low, and so normalisation of values to each animal results in a lower bias and narrower LOA and a good trending ability, which clearly demonstrates the potential of the technique. From a practical perspective, when monitoring patients in many clinical settings absolute values only offer limited information, whereas the temporal variation in values (e.g. the % change in cardiac output) from what is ‘normal’ for each individual patient provides greater clinical insight.^34^ Indeed, polar plot analysis reveals an even smaller angular bias when $\Delta {\dot{Q}}_{\text{IST}}$ and $\Delta {\dot{Q}}_{T}$ are presented as percentage changes.

Bland–Altman analysis of $\Delta \dot{Q}$ from baseline also shows the existence of greater degrees of ${\dot{Q}}_{\text{IST}}$ underestimation with higher mean $\Delta \dot{Q}$. Indeed, it is likely that the LOA at these higher $\Delta \dot{Q}$ is underestimated. This scaling error could be a consequence of the recirculation of the N_2_O sinewave emerging at higher cardiac outputs and so conflicts with assumptions made by the lung model. Clearly, further work and adjustments to the model are needed to improve the trending (and absolute agreement) of ${\dot{Q}}_{\text{IST}}$ *vs* ${\dot{Q}}_{T}$ and, as a first step, simulations using single and multi-compartmental tidal lung models are being developed to investigate these questions.

Data from the IST measurements are averaged over a 3 min rolling window and updated to the monitor every 30 s. The time of 3 min was chosen because it captures three full sinewave periods and gives robust solutions, although a shorter period could be used depending on the degree of stability required. Theoretically this approach has two potential clinical consequences. The first is that it will dampen the dynamic component of the cardiac output time series, and the second is that it results in a lag between real-time cardiac output changes and their registration. Given that even under the most challenging clinical circumstances (other than sudden cardiac arrest), cardiac output changes over a time course of minutes and the ultra-dynamic component of the time series is small; so the 3 min moving average should have so significant impact. In contrast, the ‘lag’ effect will clearly result in a delay in the registration of real-time CO values. In the data presented here, this was measured as being approximately 60 s (cf. contemporaneously measured ${\dot{Q}}_{T}$). This property is common to many other similar monitors where averaging is used (Lidco, Deltex) and needs to be taken into consideration.

The current experiment collected data from mechanically ventilated animals and so may not necessarily apply to human participants or those spontaneously breathing. Previous work has shown that IST measures of cardiopulmonary function are both accurate and reproducible in young healthy volunteers,^15^ but further work is necessary to validate the technique in human patients/participants by comparing the trending ability of ${\dot{Q}}_{\text{IST}}$ with an appropriate reference method during haemodynamic changes.

There are several limitations with the statistical approaches used in the current study. The exclusion zone in the four-quadrant plot and polar analysis aims to remove small $\Delta \dot{Q}$ which may be attributed to noise, but its definition (e.g. an absolute value or % change) is rather arbitrary. Furthermore, the polar analysis exclusion zone may inadvertently remove the most discordant measurements, that is those that have similar absolute values but in opposite directions.^18^ The concordance rate calculation in the four-quadrant plot does not account for the range of variation as, for example, a plot will be still be considered concordant if one method records $\Delta \dot{Q}$ of 100% and the other only 1%.^18^ The TIM^23^ may redress some of the limitations; however, as four-quadrant plots and polar analysis are the most commonly used approaches, and we want to compare the performance of IST with other techniques, they form the basis of our data analysis.

As with other respiratory-based approaches, the presence of lung disease or injury will likely impair aspects of ${\dot{Q}}_{\text{IST}}$ performance. The saline lavage model of acute lung injury in the current investigation reduced the trending ability of ${\dot{Q}}_{\text{IST}}$ and the concordance from a four-quadrant plot became slightly poorer. The reduced slope clearly revealed that impairments in trending ability were the result of underestimations in $\Delta {\dot{Q}}_{\text{IST}}$ relative to $\Delta {\dot{Q}}_{T}$. Any cardiac output that bypasses ventilated regions of the lung cannot be quantified by approaches relying on gas exchange measurements, and we have shown that the impaired trending of $\Delta {\dot{Q}}_{\text{IST}}$ was significantly correlated with shunt fraction. Further refinement of the lung model is required to address this issue; nevertheless, the estimation of ‘effective’ cardiac output—that is, blood flow that participates in pulmonary gas exchange—by respiratory-based techniques might also provide important clinical information in the critical care setting. For example, as increases in ${\dot{Q}}_{\text{IST}}$ should improve oxygenation and CO_2_ elimination independently of any change in cardiac output, ${\dot{Q}}_{\text{IST}}$ might provide important additional information in the optimisation of ventilator settings.

## Authors' contributions

Conception of the protocol: AF.

Design of the protocol: RB, PP, AF.

Acquisition of data: RB, DC, AM, FF, PP, AF.

Analysis and interpretation of the data: all authors.

Development of the new technique: RB, MT, PP.

Writing paper: RB.

Contributed to the production of the manuscript: DC, AM, MT, FF, PP, AF.
