# Supplementary material for: Noninvasive cardiac output monitoring in a porcine model using the inspired sinewave technique: a proof-of-concept study
Source: Br J Anaesth. 2019 Apr 4;123(2):126–34. doi: 10.1016/j.bja.2019.02.025 (PMC6676057; doi:10.1016/j.bja.2019.02.025)
Supplement: Multimedia component 1 [file mmc1.docx]

**NON-INVASIVE MONITORING OF CARDIAC OUTPUT USING THE INSPIRED SINEWAVE TECHNIQUE**

**Supplementary material**


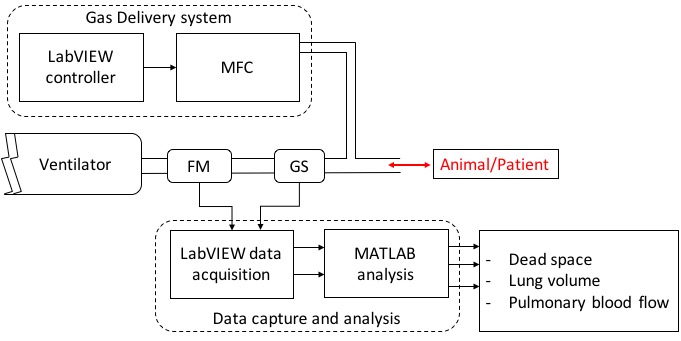


Figure 1S. Schematic representation of the Inspired Sinewave Device. Air is inhaled and exhaled through the flow meter (FM) and gas sensor (GS). Small volumes of N_2_O are delivered into inspired gas at the beginning of each inspiration via a mass flow controller (MFC). Flow and gas concentration data are recorded, and cardiorespiratory variables are recovered using a mathematical model of the lung.

**Mathematical principles of the IST**

The oscillating inspired N_2_O concentration can be defined by the equation:

$$F_{I}\left( t \right)= \bar{F_{I}}+\Delta F_{I}\sin\left( \frac{2\pi}{T}t+\phi\right) (eq.1)$$

where $\bar{F_{I}}$ is the mean amplitude, $\Delta F_{I}$ is the oscillation amplitude, $T$ is the time period (60 seconds) and $\phi$ is the phase shift.

Figure 2S illustrates the lung model used for parameter recovery. The lung consists of one dead space compartment (V_D_) and one lung compartment V_A_(t). The body is also considered as one compartment. The total pulmonary capillary blood flow through the lung is Q̇_P_(t). N_2_O tracer gas is inhaled through the dead-space into the lung and diffuses into pulmonary blood. After being distributed to different body compartments, the tracer gas renters the pulmonary circulation in mixed venous blood. It passes through dead space, before being exhaled.


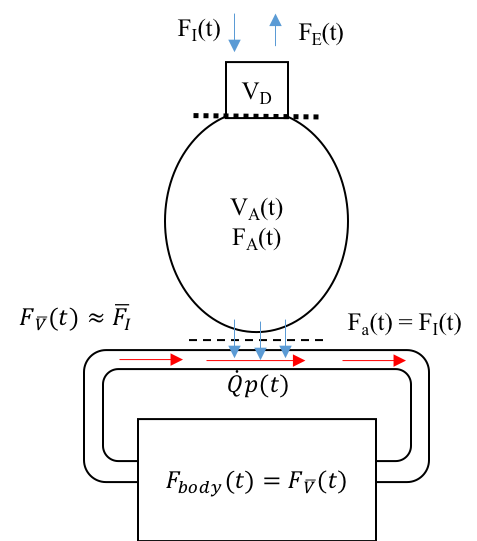


Figure 2S: The single compartment “balloon and straw” model of the lung and circulatory system

During the IST test, the F_I_(t) follows the sinewave pattern described by equation (eq.1). After an initial transient period, the alveolar concentration F_A_(t), the arterial concentration F_a_(t) and the mixed venous concentration $F_{\bar{V}}\left( t \right)$ all reach steady-state and follow sinusoidal patterns. The mixed venous concentration can then be described as:

$$F_{\bar{v}}\left( t \right)= \bar{F_{I}}+\Delta F_{\bar{v}}\sin\left( \frac{2\pi}{T}t+\phi_{\bar{v}} \right) (eq.2)$$

From Figure 2S, the mass balance of the N_2_O in the lung can be writen as:

$$Change of mass in lung = mass inhaled - mass exhaled - mass exchanged with the blood$$

So, the mass balance equation of the tracer gas of two consecutive breaths (*n*-1 and *n*) is:

$$F_{E,n-1}\times V_{A}+F_{\overline{I},n}\times\left( V_{T,n}-V_{D} \right)+F_{E,n-1}\times V_{D}-\lambda\times\dot{Q}_{P}\times\left( F_{E,n}-F_{\overline{v}} \right)\times{\Delta t}_{n} =V_{A}\times F_{E,n}+V_{T,n}\times F_{E,n} (eq.3)$$

in which:

$V_{A}$: the alveolar lung volume;

$\dot{Q}_{P}$: the pulmonary blood flow;

$V_{D}$: the deadspace, estimated from the Bohr method;

$F_{\overline{I},n}$: the mean inspired concentration of breath n^th^;

$F_{E,n-1}$,$F_{E,n}$: the end expired concentrations of breath (n-1) and n;

$\lambda$: the solubility of N_2_O, 0.47;

$F_{\overline{v}}$: the mixed venous concentration, assumed to equal to the mean of the inspired sinewave concentration at steady state $F_{I}^{0}$;

${\Delta t}_{n}$: the duration of breath n^th^;

$V_{t,n}$: the tidal volume of breath n^th^.

The mixed-venous concentration, $F_{\overline{v}}$, is assumed to be heavily damped by the body compartments and so to oscillate only negligibly at periods less than 3 minutes. Therefore, it can be considered as a constant $F_{\bar{v}}$.which approximates to the mean inspired concentration, $\bar{F_{I}}$. The mass balance equation then becomes:

$$\Leftrightarrow V_{A}\times\left( F_{E,n}-F_{E,n-1} \right)+\lambda\times\dot{Q}_{P}\times\left( F_{E,n}-\bar{F_{I}} \right)\times{\Delta t}_{n}$$

$$=V_{D}\times\left( F_{E,n-1}-F_{\overline{I},n} \right)+V_{T,n}\times\left( F_{\overline{I},n}-F_{E,n} \right) (eq. 4)$$

The volume of dead-space was calculated using Bohr method:

$$V_{D}= V_{T} \frac{F_{E}- F_{\bar{E}}}{F_{E}- \bar{F_{I}}}$$

where $F_{\bar{E}}$ is the average expired tracer gas concentration and $\bar{F_{I}}$ is the mean inspired concentration. A modified Bohr method has also been proposed to further improve the accuracy and repeatability of the airway dead space estimation^17^. This method also increases the accuracy of Q_P_ and V_A_ estimation. With $V_{D}$ and all the other parameters determined, effective lung volume (ELV = V_D_ + V_A_) and Q̇_P_ (i.e. Q̇_IST_) are estimated by solving a set of mass balance equations (eq.4).

**SUPPLEMENTARY FIGURES**


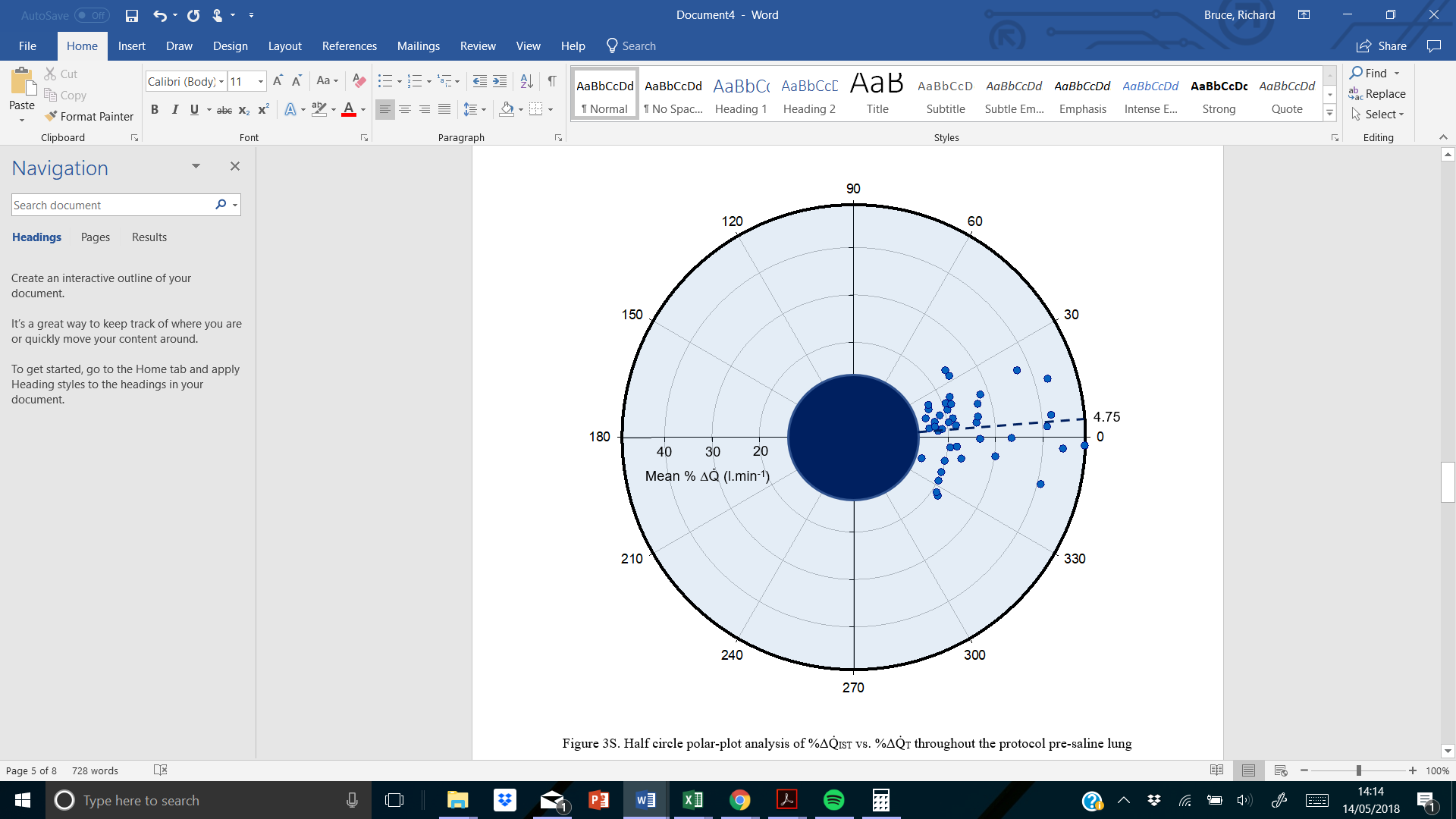


Figure 3S. Half circle polar-plot analysis of %ΔQ̇_IST_ vs. %ΔQ̇_T_ throughout the protocol pre-saline lung lavage, with an exclusion zone of 15% mean Q̇. Mean angular bias = 4.75° (-26.8°, 36.3°). Data points located within ±30° limits were considered concordant (90.5%). Data points distributed near the polar axis (0°) indicate good trending.

**SALINE LAVAGE MODEL TABLES/FIGURES**

| **Injured** |  |  |  |  |  |  |  |  |  |
| --- | --- | --- | --- | --- | --- | --- | --- | --- | --- |
| **Parameter** | **Animal Number** | | | | | | | | **Mean** |
|  | **1** | **2** | **3** | **4** | **5** | **6** | **7** | **8** |  |
| Weight (kg) | 46.5 | 45 | 39 | 53 | 37 | 37 | 47 | 44.5 | **43.0** |
|  |  |  |  |  |  |  |  |  |  |
| HR | 78 | 73 | 84 | 72 | 104 | 96 | 78 | 69 | **81** |
| SBP (mmHg) | 94 | 105 | 97 | 70 | 77 | 84 | 103 | 15 | **83** |
| DBP (mmHg) | 69 | 47 | 72 | 46 | 40 | 54 | 59 | 47 | **54** |
| Q̇_T_ (l min^-1^) | 2.7 | 7.2 | 5.6 | 3.5 | 5.2 | 4.1 | 4.1 | 3.3 | **4.5** |
| Q̇_IST_ (l min^-1^) | 2.8 | 6.6 | 6.1 | 3.2 | 4.7 | 4.6 | 6.7 | 9.1 | **5.5** |
| PAP (mmHg) | 37 | 27 | 27 | 35 | 21 | 32 | 24 | 29 | **29.1** |
| Hb (g dl^-1^) | 7.2 | 6.7 | 7.7 | 8.3 | 8 | 6.9 | 7.6 | 8.8 | **7.6** |
|  |  |  |  |  |  |  |  |  |  |
| FiO_2_ | 0.6 | 0.7 | 0.7 | 0.8 | 0.7 | 0.7 | 0.9 | 0.9 | **0.8** |
| SaO_2_ (%) | 93 | 99 | 100 | 93 | 100 | 99 | 99 | 100 | **98** |
| pH | 7.17 | 7.33 | 7.25 | 7.39 | 7.19 | 7.21 | 7.23 | 7.15 | **7.23** |
| PaO_2_ (mmHg) | 92 | 178 | 232 | 93 | 249 | 169 | 221 | 298 | **193** |
| PaCO_2_ (mmHg) | 78 | 50 | 69 | 80 | 78 | 73 | 73 | 85 | **73** |
| PFR | 153 | 255 | 331 | 116 | 355 | 241 | 245 | 331 | **250** |
| Paw Peak (cmH_2_0) | 38 | 28 | 26 | 39 | 18 | 28 | 26 | 33 | **29** |

Table 2. Baseline and respiratory and haemodynamic variables from each animal following repeated saline lavages. HR = heart rate, SBP = systolic blood pressure, DBP = diastolic blood pressure, Q̇_T_ = cardiac output from PAC thermodilution, Q̇_IST_ = cardiac output from IST; PAP = mean pulmonary artery pressure, Hb = haemoglobin, F_I_O_2_ = fraction of inspired O_2_, SaO_2_ = arterial oxygen saturation, PaO_2_ = arterial O_2_ partial pressure, PaCO_2_ = arterial CO_2_ partial pressure, PFR = PaO_2_:FiO_2_ ratio, Paw Peak = peak airway pressure. * Difference between baseline Q̇_IST_ and Q̇_T_ was 1.0 l.min^-1^ (-1.1, 3.1), *P* = 0.28).


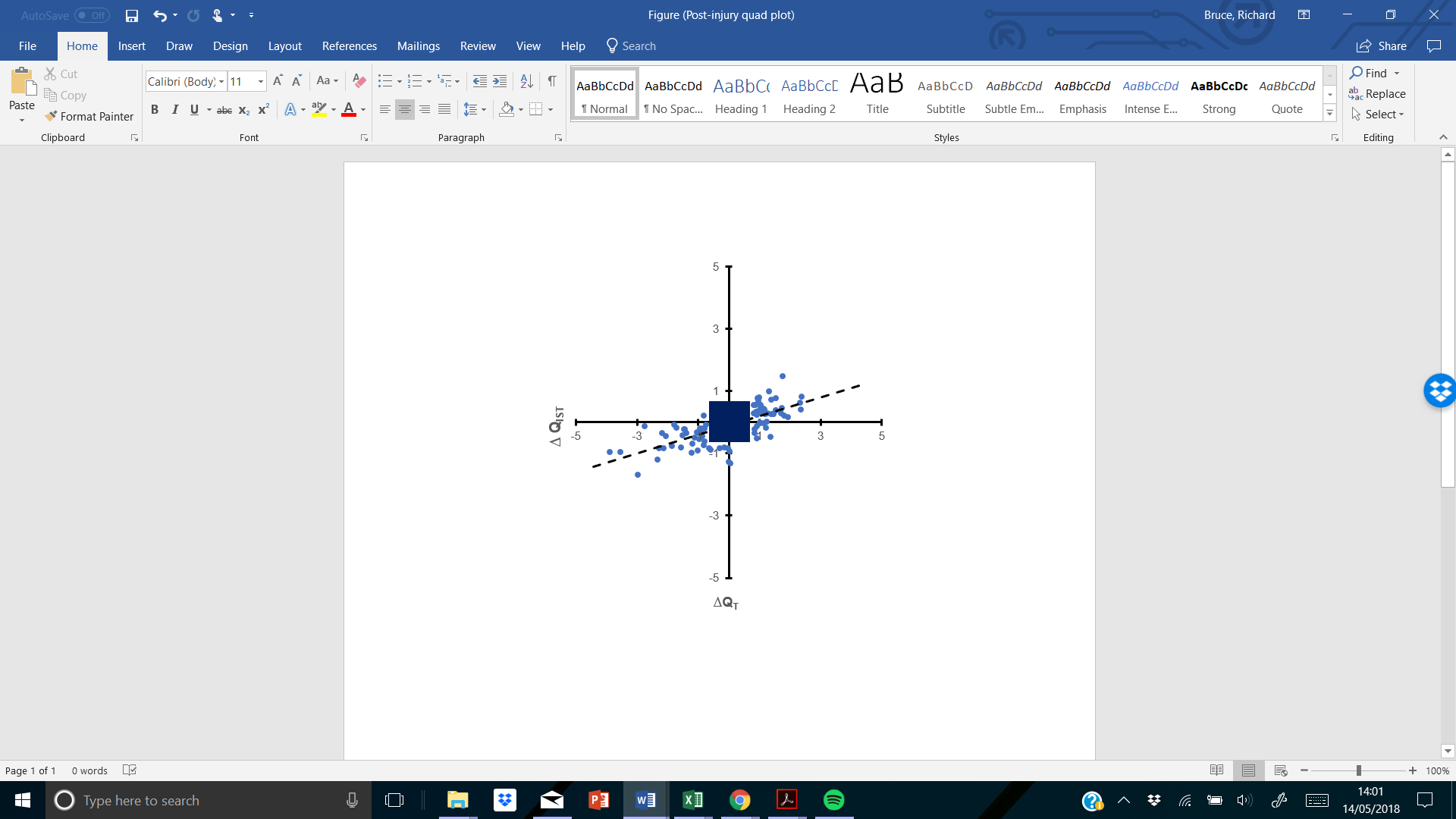


Figure 4S. Four-quadrant plot analysis of ∆Q̇IST vs. ∆Q̇T throughout the protocol post saline lung lavage, with an exclusion zone of 15% mean Q̇_T_ (0.75 l min^-1^). Linear regression analysis reveals an equation of ∆Q̇_IST_ 0.3 x ∆Q̇_T_ + 0.1, with r = 0.72. Data points located in either quadrant of agreement were considered concordant (89.4%)


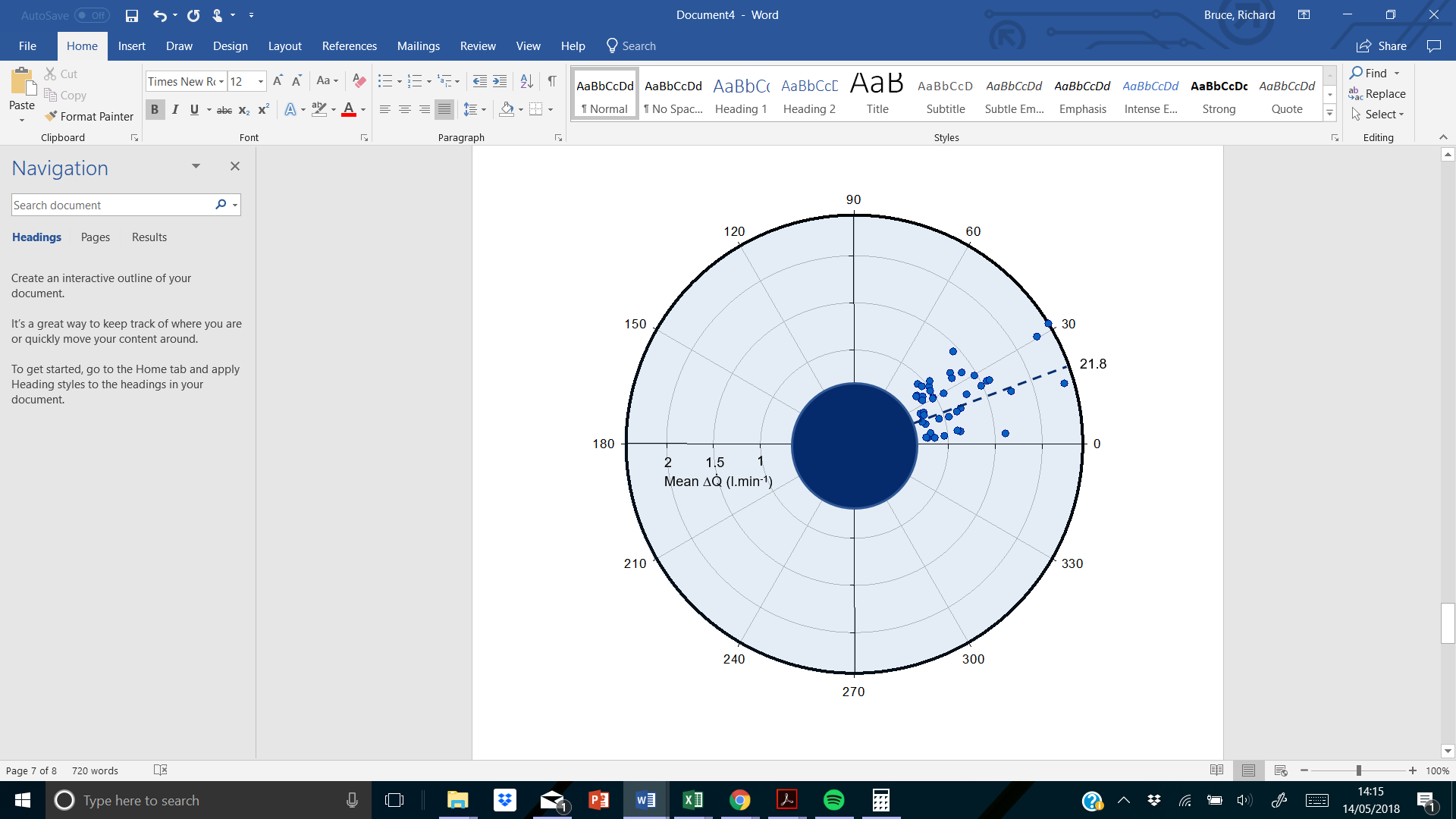


Figure 5S. Half circle polar-plot analysis of %ΔQ̇_IST_ vs. %ΔQ̇_T_ throughout the protocol post-saline lung lavage, with an exclusion zone of 15% mean Q̇_T_ (0.75 l.min^-1^). Mean angular bias = 21.8° (-4.2°, 47.6°) Data points distributed near the polar axis (0°) indicate good trending.


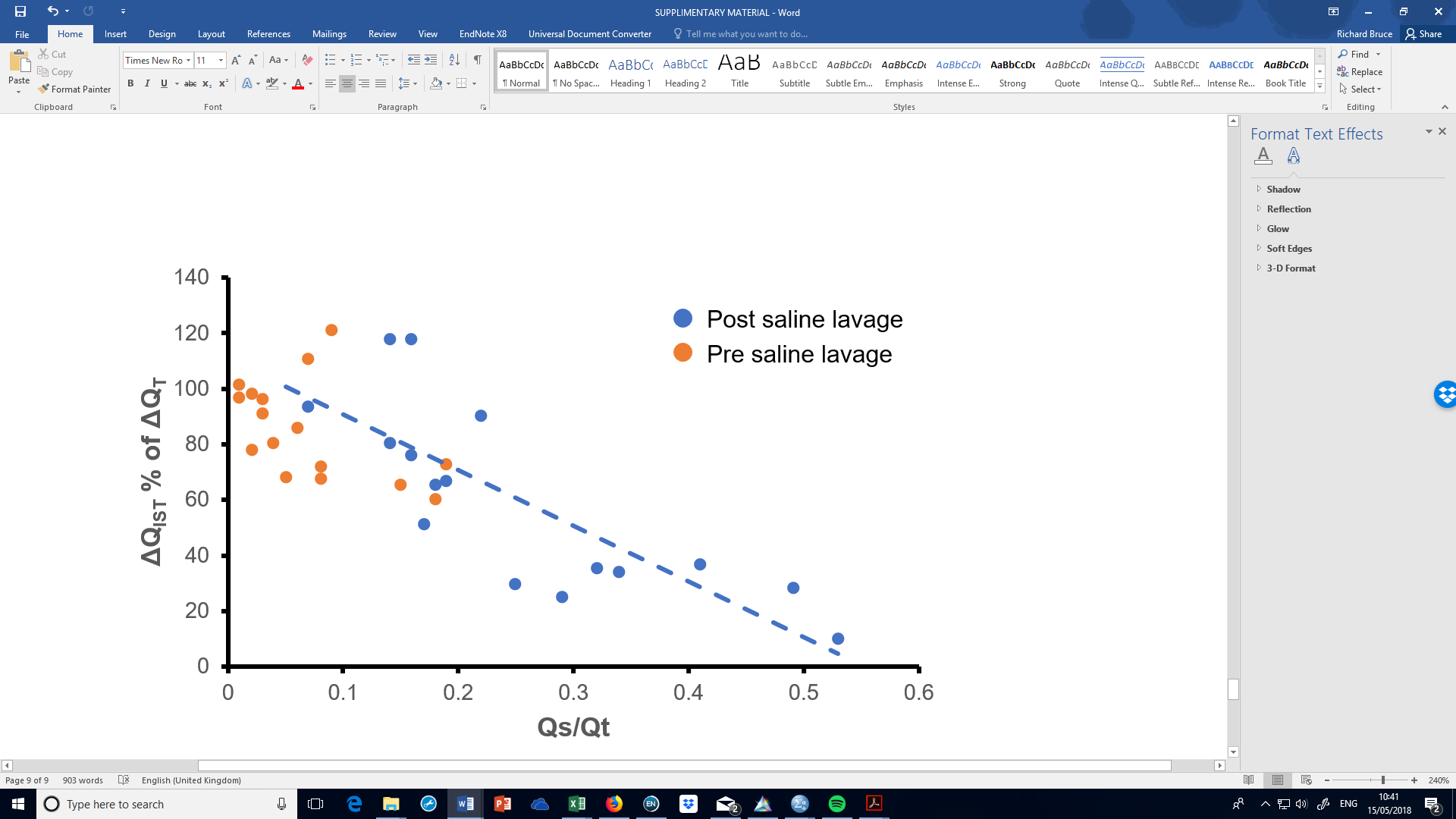


Figure 6S. The correlation, between shunt fraction (Q̇s/Q̇t) and the change in Q̇_IST_ expressed as a percentage of the change in Q̇_T_ (ΔQ̇_IST_ % of ΔQ̇_T_). Orange dots = pre-lavage; Blue dots = Post lavage; r = 0.79, *P*<0.05. Arterial blood gas samples, needed for the calculation of Q̇s/Q̇t, were collected on two occasions during each protocol: 1) during the nadir of Q̇_T_ and 2) during peak Q̇_T_. As such, ΔQ̇_IST_ and ΔQ̇_T_ were calculated as 1) the change in Q̇ from baseline to nadir and 2) the change in Q̇ from nadir to peak.


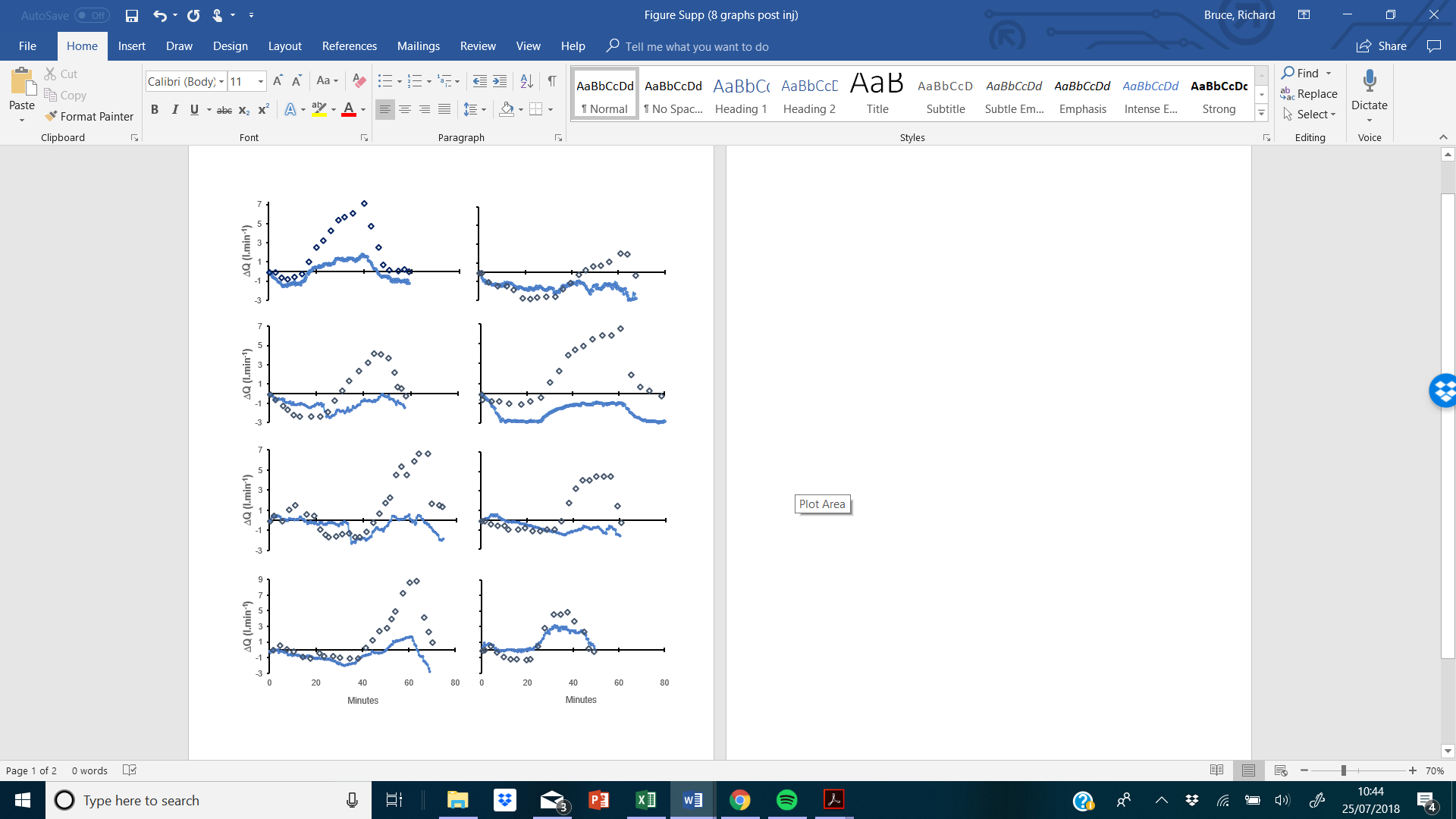


**H**

**G**

**F**

**E**

**D**

**C**

**B**

**A**

Figure 7S A-H. ΔQ̇ from baseline in all 8 animals throughout the protocol. Dark blue diamonds are Q̇_T_ measurements, and light blue dots and lines are Q̇_IST_ measurements.
